# Targeting Signal 3 Extracellularly and Intracellularly in Graft-Versus-Host Disease

**DOI:** 10.3389/fimmu.2020.00722

**Published:** 2020-04-28

**Authors:** Stephanie Kim, Pavan Reddy

**Affiliations:** ^1^Division of Hematology and Oncology, Department of Internal Medicine, University of Michigan, Ann Arbor, MI, United States; ^2^Medical Scientist Training Program, University of Michigan, Ann Arbor, MI, United States

**Keywords:** bone marrow transplantation, graft-versus-host disease, cytokines, alloimmunity, intracellular trafficking

## Abstract

Allogeneic hematopoietic stem cell transplantation (allo-HCT) holds curative potential for many hematological disorders. However, the pathophysiology of the desired graft-versus-tumor effect is linked to life-threatening complications of acute graft-versus-host disease (GVHD). Allogeneic donor T lymphocytes are essential for causing GVHD, and their activation relies on the coordination of TCR engagement and co-stimulation, also known as Signal 1 and Signal 2. In addition to these signals, a network of secreted cytokines by immune cells provides a third signal, Signal 3, that is critical for the initiation and maintenance of GVHD. Strategies to target Signal 3 in human diseases have shown therapeutic benefit for inflammatory disorders such as Rheumatoid Arthritis and Inflammatory Bowel Disease. However, despite our growing understanding of their role in GVHD, the success of targeting individual cytokines has been modest with some notable exceptions. This review aims to describe current approaches toward targeting Signal 3 in clinical GVHD, and to highlight emerging studies in immune cell biology that may be harnessed for better clinical translation.

## Introduction

Acute Graft-versus-Host Disease (GVHD) is a major cause of non-relapse morbidity and mortality in patients receiving allo-HCT. While the development of GVHD is dependent on numerous factors, the HLA major and minor antigen-induced activation of donor-derived T cells is the key determinant for induction and severity of GVHD (1). T cell activation occurs as a result of the engagement of three signals (2, 3). Signal 1 is provided by the T cell receptor with cognate peptide:HLA, and Signal 2 follows the engagement of T cell co-stimulatory receptors by cognate ligands on antigen presenting cells (2). Interaction with both antigen and co-stimulatory ligands is critical for initiating the intracellular signaling cascade that promotes T cell proliferation, survival, and effector functions, and therefore a number of approaches aim to target these steps in the prevention and treatment of GVHD (4–6). In addition to these two signals, Signal 3 which is provided by surrounding cytokines controls the differentiation of T helper (Th) subsets, influences the polarization of specific T effector responses, and shapes the balance between immune activation and tolerance (3, 7, 8).

The cytokine milieu in patients following bone marrow transplantation is complex, and includes both immune cell and target tissue sources. It is released following conditioning treatments and amplified by tissue destruction from T cell-mediated lysis (9). In both experimental models of allo-HCT and patients affected by GVHD, the cytokines that provide Signal 3 significantly impact the alloreactive T cell response (10). Selectively targeting the cytokines that promote alloreactive T cells is therefore an attractive therapeutic strategy.

Many existing therapies for GVHD are aimed at targeting donor T cells or inflammatory byproducts of immune cells which contribute to symptoms and pathology. However, donor T cells are also critical for the graft-versus-tumor (GVT) effect, and as such, balancing therapies has been a challenge to preserve sufficient GVT activity while minimizing GVHD-related tissue damage. One strategy has been to target the cytokines that promote alloreactive T cell toxicity and also cause direct inflammation-related organ damage. While the success of these approaches has been modest thus far, a growing basic science understanding of relevant cytokines, the regulation of cytokine secretion, and the specific impact each has on immune and target cells will inform future strategies for the prevention and treatment of GVHD.

**FIGURE 1:**
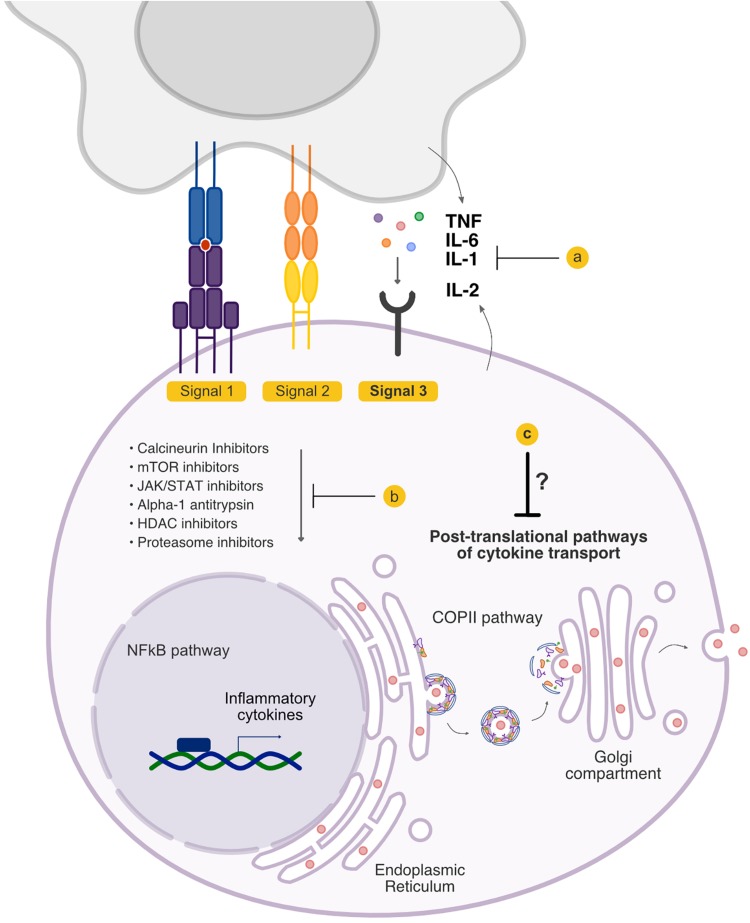
Signal 3 pathways that are targeted in graft-versus-host disease (GVHD). Signal 3 critically shapes the allo-response, and existing treatment options have the potential to modulate the cytokine milieu that accompanies allogeneic T cell activation. Current treatment strategies include **(a)** blockade of cytokines extracellularly and blockade of cytokine receptors, and **(b)** inhibition of the downstream signaling cascades that culminate in the production of inflammatory cytokines. **(c)** The pathways that regulate cytokine secretion following their synthesis but preceding their release have not been therapeutically targeted.

Several outstanding recent articles have reviewed the biology and the important role of cytokines in both acute and chronic GVHD (10–13). In this review, we will only highlight cytokines that serve as Signal 3 to T cells (Figure 1) and have been targeted in clinical acute GVHD. Specifically, we will first briefly review approaches that directly target Signal 3 secreted cytokines, the majority of which have been targeted upon their release extracellularly (i.e., post-synthesis and release, in the extracellular space). We will then focus on therapies that target the induction of cytokine synthesis intracellularly in immune cells, focusing on specific cell signaling pathways that lead to cytokine synthesis (i.e., pre-synthesis by targeting intracellular signaling cascades). Finally, we will review an as yet understudied area to target the cytokines following their synthesis intracellularly, including post-translational pathways, and the intracellular trafficking pathways that regulate their release. We will discuss why understanding the pathways by which these cytokines are transported intracellularly may represent an effective approach toward the rational design of GVHD therapies.

## Direct T Cell Intrinsic Cytokines and Proliferative Responses

Strategies to control donor T cell activity begin with broadly acting anti-inflammatory prophylactic agents. The most widely used approaches today include methotrexate in combination with cyclosporine or tacrolimus. Methotrexate, a folate antagonist, can target rapidly proliferating allogeneic T cells and be cytotoxic to their growth. Cyclosporine and Tacrolimus inhibit the calcineurin-dependent activation of NFAT transcription factors and their translocation from the cytoplasm to the nucleus, reducing the transcription of inflammatory cytokines such as IL-2 and IFNγ by T cells. Targeting the immune response at this level inhibits key T effector functions as well as their proliferation.

While instrumental in reducing GVHD risk, however, standard prophylaxis measures are not completely effective in preventing the onset of GVHD. Furthermore, they confer non-specific anti-inflammatory functions that can increase the risk of tumor relapse and infection. Systemic glucocorticoids which remain the mainstay of first-line treatment of acute GVHD is also broadly immunosuppressive. However, T cells are responsive to the influence of select cytokine signals which promote their growth, proliferation, cytotoxicity, and secretion of effector molecules. Important signals include cytokines that promote inflammation and which also tend to be increased following conditioning and allo-HCT such as IL-12, IL-4, IL-1, TNFα, and IL-6 (14, 15). Therefore, agents that attenuate the cytokine signals that promote the overactivity of T cells could be beneficial in GVHD treatment. While all of these cytokines have been shown to be critical sources of Signal 3, agents that block TNF, IL-6, and IL-1, as well as the T cell-derived growth factor IL-2 have been studied as potential modes of treatment in acute GVHD. There remains active interest in the use of specific anti-inflammatory cytokine blockade including agents that directly target activating cytokine signals to T cells extracellularly.

## Extracellular Signal 3 Blockade

### Anti-TNF

Tumor Necrosis Factor (TNF) is a cytokine that acts on multiple immune cell types, and promotes the production of other Signal 3 cytokines including IL-1 and IL-6 (16). Overexpression of TNF is implicated in the development of multiple autoimmune and inflammatory disorders, and its blockade has improved disease management and quality of life for patients with rheumatoid arthritis, inflammatory bowel disease, and others (17). In addition to innate immune cell-derived TNFα, T cell-derived TNFα is implicated in the development of GVHD in experimental murine models, and in humans, it has been observed that early increased serum levels of TNFα are associated with major transplant-related complications (18, 19). TNFα levels are initially increased following conditioning treatments as measured by levels of TNF receptor-1 which correlate with those of plasma TNFα (20, 21). TNFα is appreciated to play direct roles in acute GVHD pathogenesis by both effecting direct tissue damage and by promoting allogeneic T cell cytotoxicity (22). Therefore, the use of neutralizing anti-TNFα agents has been studied for efficacy in GVHD prevention and treatment. One such agent is infliximab, an anti-TNFα monoclonal antibody that has been studied in prophylaxis, and treatment of steroid-refractory acute GVHD (SR-aGVHD) (23, 24). However, in patients with SR-aGVHD, the addition of infliximab did not improve survival and instead increased post-transplant risk of infection when compared to treatment with corticosteroids alone (24, 25). Clinical trials have also been performed to test the efficacy of etanercept for the blockade of soluble TNF. However, early results were not borne out by a multi-center randomized study that demonstrated no impact of etanercept on GVHD or on overall survival, infection, and relapse of the primary malignancy (26–30). While TNFα is best known for its inflammatory properties in promoting T cell effector responses, its dual role as a suppressive cytokine is increasingly being appreciated with the characterization of functionally disparate TNF receptors and their actions on different cell types such as T regulatory cells (Tregs) (31). While the vast majority of current therapies target TNF directly, future studies of specific inhibitors of TNF production, signaling, and oligomerization, may clarify their potential in the treatment of GVHD (17).

### Anti-IL-6

IL-6 is a member of a family of cytokines that shares the receptor complex gp130, a widely expressed signaling complex that leads to the activation of associated Janus kinase (JAK) and signal transducer and activation of transcription (STAT) pathways. Levels of circulating IL-6 can increase dramatically in settings of inflammation, and consequently, IL-6 is associated with the acute phase inflammatory response (32). The biological consequences of IL-6 are wide-ranging and include the pathologic stimulation of proinflammatory responses, such as by promoting Th17 cell development and inhibition of regulatory T cell differentiation (33). In murine models of GVHD, donor T cell-derived IL-6 critically contributes to disease severity, and donor T cell-specific deficiency of IL-6 decreases GVHD-related mortality (34). Blockade of the IL-6 signaling in experimental models also improved GVHD survival and led to an increase in regulatory T cells, and decreased Th1 and Th17 cells in target organs while preserving GVT effects (34, 35). A clinical study examining the effect of tocilizumab, a humanized monoclonal antibody against the IL-6 receptor, showed favorable outcomes in a small number of patients with either acute or chronic GVHD (36). A phase I/II clinical trial further studied the effect tocilizumab administered 1 day prior to allogeneic peripheral blood stem cell transplantation in patients that received cyclosporine and methotrexate as GVHD prophylaxis (37). A phase II trial in which patients received busulfan-based conditioning prior to receiving tocilizumab with tacrolimus and methotrexate showed a low incidence of gastrointestinal GVHD (38). Recently, a phase III randomized and double-blinded trial observed a trend toward a reduced overall incidence of grade II-IV GVHD in patients receiving tocilizumab, but no difference in long term survival compared to controls (39).

### Anti-IL-1

In addition to TNF and IL-6, IL-1 is an inflammatory cytokine that is increased following conditioning and is critical for immune homeostasis, but when dysregulated, potentiates GVHD pathology (10). Although small early studies showed that targeting soluble IL-1 with a recombinant human IL-1 receptor or administration of a recombinant IL-1 receptor antagonist could ameliorated SR-aGVHD, these results were not confirmed in a randomized controlled trial (40–42). Given its known potency in inflammatory disorders and association with GVHD in both experimental models and humans, strategies that target IL-1 may be effective depending on the phase of acute and/or chronic GVHD (43, 44). Key upstream regulators of IL-1 include intracellular immune sensors NLRP3 and NLRP6, which assemble into inflammasomes in settings of cellular damage and stress such as those induced by pre-transplant conditioning therapies. Following allogeneic transplant in experimental models, NLRP3 inflammasomes induced the secretion of pathogenic levels of IL-1β by multiple intestinal cellular sources, and also controlled the IL-1β-dependent skewing of Th17 differentiation critical to the development of GVHD (44). By contrast, donor myeloid derived suppressor cells, which have immunoregulatory functions in GVHD, can lose suppressive capacity following activation of the inflammasome (45). Thus, the cellular source is an important determinant of the impact of inflammasome dependent effects on GVHD. In host non-hematopoietic target tissues, NLRP3 inflammasomes serve a protective role in promoting intestinal epithelial cell integrity and repair by increasing IL-18 secretion (46). NLRP6, which has protective roles in intestinal colitis, plays a role in aggravating gastrointestinal GVHD when expressed in host-non-hematopoietic tissue, and its absence in host intestinal epithelial cells helps maintain gut homeostasis following allogeneic BMT in experimental models (47). It is likely that the effects mediated by NLRP6 may be IL-1 independent or dependent depending on the type of immunopathology.

### Anti-IL-2

IL-2 expression is increased upon activation, is released by T cells, and serves as a growth factor for T effector cells and Tregs. IL-2 is one of the earliest cytokines to be studied as a target for immunosuppression therapeutically (48–50). Intracellular targeting of the production and secretion of IL-2 with calcineurin inhibitors remains the first line prophylaxis strategy in the prevention of GVHD. Studies have also explored the use of monoclonal antibodies against IL-2 receptor including daclizumab, basiliximab, and inolimomab, to target the activity of secreted IL-2. One randomized trial found that the addition of daclizumab to corticosteroids as an initial therapy for acute GVHD resulted in increased GVHD-related mortality (51). A phase II study found that while treatment of SR-aGVHD with daclizumab led to an increased complete response rate, it was associated with higher rates of long-term complications of chronic GVHD (52). Although basiliximab appears to be better tolerated by patients and not associated to the same degree of adverse events in initial studies, future studies are needed to determine its safety and efficacy (53–55). Targeting IL-2 is nuanced by its dual roles, as in addition to promoting the T cell-mediated toxicity in GVHD, it is essential for the development and maintenance of Tregs which are important regulators of immune tolerance, and may in turn be employed in the prevention of GVHD (56–60). Therefore, efforts to target IL-2 must balance its inflammatory and immunoregulatory effects that minimize GVHD but still prevent relapse of the primary disease. The administration of low-dose IL-2 is of interest in the treatment of chronic GVHD, and has been associated with expansion of Tregs, suppression of conventional T cell proliferation, and long-term reduction of chronic GVHD symptoms (61–63).

## Targeting the Intracellular Afferent Arm of Signal 3 Cytokine Release

Following their activation, T cells engage distinct signaling pathways that lead to the increased synthesis of important effector molecules including cytokines and cytotoxic factors. These culminate in a pro-inflammatory milieu that shapes the allogeneic T cell response and also causes direct tissue damage. While targeting cytokines following their release by immune cells is gaining increasing interest for their promising outcomes in both experimental models and clinical trials, there has been renewed interest in targeting earlier steps following T cell activation such as the intracellular signaling pathways that increase cytokine production. Therapeutic strategies have included targeting the intracellular signaling pathways that lead to proinflammatory cytokine transcription and translation with agents such as calcineurin inhibitors as described above, mTOR inhibitors, JAK inhibitors, Alpha-1 Antitrypsin, histone deacetylase inhibitors, and proteasome inhibitors.

### mTOR Inhibition

The mammalian target of rapamycin (mTOR) pathway is a major regulator of cellular growth and metabolism that is also critical for T cell activation, differentiation, and function (64). Sirolimus, an inhibitor of mTOR, has been demonstrated to exhibit anti-inflammatory effects through multiple mechanisms including inhibition of both conventional T cell and dendritic cell activity, and promotion of Treg development (65–67). Early studies showed that sirolimus can be well tolerated in patients and may be associated with a lower risk of GVHD (68, 69). A prospective randomized trial found that in combination with tacrolimus, sirolimus is a safe alternative to cyclosporine and methotrexate for GVHD prophylaxis (70). A recent phase III trial reported that the addition of sirolimus to cyclosporine and mycophenolate mofetil for prophylaxis showed efficacy in lowering the incidence of GVHD (71). However, its efficacy as a therapy for SR-aGVHD in combination with other agents may be limited depending on the stage of GVHD, and warrants further studies (72).

### JAK1/2 Inhibition

T cells are responsive to inflammatory cytokines including IL-6 and interferons via their propagation of JAK/STAT pathways. Activation of the JAK family of proteins leads to the phosphorylation of STATs, which translocate to the nucleus and are critical regulators of T cell alloreactivity (73). Pre-clinical models demonstrated that targeting JAK1/2 targets GVHD but preserves GVL, with the contribution of decreased serum levels of proinflammatory cytokines including IL-6 (74–76). This led to testing the effects of JAK inhibitors such as ruxolitinib, baricitinib, and itacitinib. Ruxolitinib, a selective inhibitor of JAK1/2, in patients with SR-aGVHD. In an early study, six patients experienced reduced GVHD in correlation with a decrease in proinflammatory cytokines in the serum (75). Additional clinical trials are underway to examine the effects of ruxolitinib in patients with SR-aGVHD (77). Itacitinib, a selective JAK1 inhibitor, has also demonstrated safety in a phase I trial and studies of its efficacy in the treatment of SR-aGVHD are ongoing (78).

### Alpha-1 Antitrypsin

Alpha-1 Antitrypsin (AAT) is an endogenously circulating serine protease inhibitor that, when deficient or mutated, has been described in the pathogenesis of disorders including COPD, cirrhosis, and multiple neurodegenerative diseases (79). In addition, AAT has a suppressive role in inflammatory settings with an appreciable inhibitory effect on TNF levels (80, 81). When AAT is administered in models of murine allo-HCT, it has been shown to reduce GVHD-induced mortality while preserving the allogeneic T cell GVL effect (82–84). The therapeutic benefit in these models has been linked to a decrease in alloreactive effector T cells and inflammatory cytokines, and an increase in Tregs and immunoregulatory cytokines such as IL-10 (84). AAT is an effective modulator of the profile of circulating cytokines following allo-HCT leading to significantly reduced disease murine models, underscoring the therapeutic potential of AAT and strengthening the rationale for studying the effect of AAT therapy in humans. Recent studies showed complete recovery in 4 of 12 patients and improvement in the other 8 patients with SR-aGVHD (85). A prospective multi-center study that followed tested AAT as a first line therapy for SR-aGVHD led to an overall response rate of 65% and complete response rate of 35% by day 28 (86). Ratios of T effector cells and Tregs were consistent with those observed in experimental models (86). Both studies found that AAT is well tolerated by patients, is not associated with an excessive risk of infection, and are now being studied in a randomized manner in a phase III study.

### Histone Deacetylase Inhibition

Histone deacetylase (HDAC) inhibitors represent a diverse class of drugs that cause reversible inhibition of HDAC enzymes, remodel chromatin structure, and differentially modify gene expression depending on the specific HDAC, cell type, and context. A clinically significant consideration of HDAC inhibitors is that in addition to acting on histones, they can have non-specific effects on other protein deacetylases that broadly regulate cell growth and signaling (87). However, at non-cytotoxic doses, HDAC inhibitors have recently been appreciated to be well tolerated and exhibit immunoregulatory properties, lending to growing interest in their potential to treat inflammatory diseases (88). Among their diverse effects, HDAC inhibitors have shown immunomodulatory effects on dendritic cell and macrophage antigen presentation, TLR pathways, and IFN signaling (88). As a consequence, they can reduce the expression of cytokines involved in Th1 and Th17 differentiation such as IL-6 and IL-12 (89, 90). In experimental models of GVHD, HDAC inhibition has been observed to lead to reduced secretion of proinflammatory cytokines including IL-12, IL-6, and TNFα by dendritic cells through enhancing the expression of indoleamine 2,3 dioxygenase (91–93). Two phase II clinical trials have examined oral HDAC inhibitor vorinostat in the prevention of GVHD. One study investigated the addition of vorinostat to tacrolimus and mycophenolate in patients that received reduced intensity conditioning prior to related donor hematopoietic stem cell transplantation (94). Another study tested the effect of vorinostat when combined with tacrolimus and methotrexate following myeloablative conditioning prior to unrelated donor allo-HCT (95). Both studies showed that vorinostat is well tolerated and associated with a lower incidence of acute GVHD (94, 95). A third study is ongoing to evaluate vorinostat as preventive therapy in adolescents and young adults receiving allogeneic BMT when combined with standard preventive therapy (NCT03842696). Future studies will elucidate the clinical benefit of HDAC inhibitors including vorinostat, as well as other agents such as panobinostat that are more recently being evaluated as primary therapy for acute GVHD (96).

### Proteasome Inhibition

The ubiquitin proteasome pathway is central to the selective of maintenance of proteins, and regulates a diverse set of intracellular processes including quality control for misfolded proteins, regulation of the cell cycle, and peptide processing for antigen presentation (97). In immune cells, the proteasome is also involved in cell signaling, notably by regulating the expression of NF-κB, a transcription factor that promotes cell survival and the expression of numerous inflammatory cytokines (98). Proteasome inhibitors have thus emerged as a drug class that is associated with a number of immunomodulatory effects, and is currently approved for the treatment of a number of hematologic disorders (99). Proteasome inhibitors have been shown suppress NF-κB activation, in part due to the reduction of proteasome-dependent degradation of IκB (100, 101). The inhibition of NF-κB is associated with reduced proliferation, survival, and toxicity of allogeneic T cells, and has also been shown to abrogate T cell cytokine production (102, 103). In addition to its effect on T cells, proteasome inhibitors such as bortezomib have suppressive effect on dendritic cell maturation and inflammatory cytokine production, while increasing dendritic cell apoptosis, highlighting their influence on multiple processes and cell types (104). In murine models of acute GVHD, treatment of recipients with bortezomib led to increased survival and protection from GVHD while maintaining GVT activity (105, 106). However, the timing of bortezomib administration may be critical in determining its efficacy as well as its overall safety, as delayed administration (i.e. 5 or more days after BMT) compared to 0 to 3 days following BMT results in increased gastrointestinal toxicity. This mechanistically correlates in other studies with amplified IL-1β production by dendritic cells (107, 108). While an early phase I/II study to test a prophylaxis regimen of bortezomib combined with tacrolimus and methotrexate showed that this combination was well tolerated and associated with a lower incidence of GVHD, a randomized controlled trial failed to show an improvement in grade II-IV acute GVHD incidence with the addition of bortezomib, compared to methotrexate and tacrolimus alone (109, 110). Another proteasome inhibitor ixazomib improves acute GVHD upon early administration, impairs dendritic cell development, cytokine production, and expression of co-stimulatory molecules consistent with reduced proliferation of T cells, and clinical trials are underway to determine its efficacy in post-transplant patients (111).

## Targeting the Intracellular Efferent Arm of Signal 3 Cytokine Release

The majority of pre-clinical studies have provided the foundation for the development of therapies that target cytokines or cytokine receptors directly, or the signaling pathways that govern their transcription and translation. A gap in knowledge remains, however, in the post-translational intracellular pathways that coordinate the transport mechanisms that regulate cytokine release by immune cells. Multiple transport steps coordinate the membrane biogenesis, transport, and fusion events that carry cytokines between intracellular compartments and toward the cell surface for secretion. Better understanding of these intracellular secretory pathways utilized by cytokines in immune cells may provide important insights into novel therapeutic targets.

### The post-Golgi Apparatus Transport of Cytokines

In the classical secretory pathway, proteins are co-translationally inserted into the endoplasmic reticulum (ER), and transported to the Golgi compartment where they undergo further processing and are delivered to other intracellular compartments, or the extracellular space. Activated T cells undergo morphologic changes that affect intracellular cytokine transport by first establishing polarity and forming immune synapses with antigen presenting cells. A dynamic cytoskeleton enables T cells to both adhere to the APC, and transport secretory vesicles containing cytokine cargoes (112). CD8^+^ T cells engaged with cognate APCs reorient their microtubule organizing center toward the immunological synapse, and transport secretory granules along microtubules toward the point of cell-cell contact for targeted lysis of the APC (113). CD4^+^ T cells remain less well characterized in their regulated secretory pathways than CD8^+^ T cells. However, distinct post-Golgi pathways have been elucidated, including a directional pathway that directs cytokines toward the immunological synapse and minimizes non-specific cytokine release, and a multi-directional pathway to promote more generalized inflammation (114). Studies to elucidate the molecular mediators of regulated T cell secretion may enable novel approaches toward controlling targeted cytokine release in disease states. These studies and others underscore that in addition to cytokine expression, regulation of the membrane-bound organelles that transport them significantly impact the consequences of T cell activation. However, to date, studies have been limited to understanding the secretory pathway of cytokines through events that occur after their egress from the Golgi apparatus.

### Targeting Early Intracellular Phases of the Efferent Arm of Signal 3 Release

About one-third of encoded proteins are estimated to be targeted to the ER and destined for the secretory pathway (115, 116). Coat Protein Complex II (COPII), a complex of five highly conserved proteins (Sec23/Sec24, Sec13/Sec31, and Sar1), assembles at the ER membrane and forms vesicles that incorporates proteins for transport to the Golgi compartment, including many secreted proteins (Figure 1). The molecular components of COPII were first described in *Saccharomyces cerevisiae* (117, 118), and COPII-mediated ER-Golgi transport is conserved in all eukaryotes including humans (119). As our understanding of the COPII-dependent secretory pathway increases, the characterization of cell- and context-specific activities and regulation of protein secretion will be critical. Fundamental gaps remain in our knowledge about the role of the early secretory pathway in specific cytokine secretion, and the relevant molecular regulators of this process by immune and other cells. Recently, we have begun to decipher the role of the COPII pathway in the release of cytokines by T cells. We observed that disrupting COPII coat formation by targeting SEC23 results in greatly reduced pathogenicity of donor T cells in experimental models of GVHD (120). Future studies on how the COPII pathway regulates secretion of critical Signal 3 cytokines may further shed light on immune cell secretory pathways and provide insight into potential novel therapeutic targets.

### Targeting the Timing of Signal 3 for Mitigating GVHD

Cytokine secretion and its downstream effects are dynamic and context dependent. Signal 3 cytokines are typically studied and understood as discussed above in the context of APC activation and induction of T cell response. The role of signal 3 in the perpetuation of an ongoing T cell response is unclear. Based on the known data the timing of targeting signal 3, it may be critical for mitigating GVHD. Specifically, given its role in induction of allogeneic T cell response, it may be more effective to target signal 3 in prevention strategies for either incidence of GVHD or in preventing steroid-refractoriness following onset of severe GVHD. However, because cytokine cascades and inflammatory responses may wax and wane, the exact timing will need to be carefully determined experimentally and in clinical studies.

## Concluding Remarks

The relevance of cytokines that serve as Signal 3 for robust T cell responses is increasingly well established in their role in promoting GVHD, and as promising therapeutic targets. However, current approaches have yielded modest success and additional strategies are warranted. Moving forward, identifying shared intracellular trafficking pathways that control cytokine release may be of value in developing newer approaches to target Signal 3. Basic science research on the fundamental and critical determinants of intracellular trafficking pathways that coordinate their release remain to be understood. With a better mechanistic understanding of these pathways, the identification of key molecular mediators in the allogeneic setting will be essential. Exploring these questions will both enhance our fundamental understanding of immune regulation, and may pave the way for controlling T cell immunity in inflammatory disorders.

## Author Contributions

SK and PR wrote and edited the manuscript.

## Conflict of Interest

The authors declare that the research was conducted in the absence of any commercial or financial relationships that could be construed as a potential conflict of interest.

## References

[1] Ferrara JL Levine JE Reddy P Holler E. Graft-versus-host disease. *Lancet.* (2009) 373:1550–61. 10.1016/s0140-6736(09)60237-3 19282026 PMC2735047

[2] Smith-Garvin JE Koretzky GA Jordan MS. T cell activation. *Annu Rev Immunol.* (2009) 27:591–619. 10.1146/annurev.immunol.021908.132706 19132916 PMC2740335

[3] Curtsinger JM Mescher MF. Inflammatory cytokines as a third signal for T cell activation. *Curr Opin Immunol.* (2010) 22:333–40. 10.1016/j.coi.2010.02.013 20363604 PMC2891062

[4] Wallace PM Johnson JS MacMaster JF Kennedy KA Gladstone P Linsley PS. CTLA4Ig treatment ameliorates the lethality of murine graft-versus-host disease across major histocompatibility complex barriers. *Transplantation.* (1994) 58:602–10. 10.1097/00007890-199409150-00013 8091487

[5] Briones J Novelli S Sierra J. T-cell costimulatory molecules in acute-graft-versus host disease: therapeutic implications. *Bone Marrow Res.* (2011) 2011:976793. 10.1155/2011/976793 22046574 PMC3195325

[6] Poirier N Mary C Dilek N Hervouet J Minault D Blancho G Preclinical efficacy and immunological safety of FR104, an antagonist anti-CD28 monovalent Fab’ antibody. *Am J Transplant.* (2012) 12:2630–40. 10.1111/j.1600-6143.2012.04164.x 22759318

[7] Curtsinger JM Lins DC Mescher MF. Signal 3 determines tolerance versus full activation of naive CD8 T cells: dissociating proliferation and development of effector function. *J Exp Med.* (2003) 197:1141–51. 10.1084/jem.20021910 12732656 PMC2193970

[8] Curtsinger JM Schmidt CS Mondino A Lins DC Kedl RM Jenkins MK Inflammatory cytokines provide a third signal for activation of naive CD4+ and CD8+ T cells. *J Immunol.* (1999) 162:3256–62. 10092777

[9] Toubai T Mathewson ND Magenau J Reddy P. Danger signals and graft-versus-host disease: current understanding and future perspectives. *Front Immunol.* (2016) 7:539. 10.3389/fimmu.2016.00539 27965667 PMC5126092

[10] Henden AS Hill GR. Cytokines in graft-versus-host disease. *J Immunol.* (2015) 194:4604–12. 10.4049/jimmunol.1500117 25934923

[11] Kumar S Mohammadpour H Cao X. Targeting cytokines in GVHD therapy. *J Immunol Res Ther.* (2017) 2:90–9. 28819653 PMC5557058

[12] MacDonald KP Blazar BR Hill GR. Cytokine mediators of chronic graft-versus-host disease. *J Clin Invest.* (2017) 127:2452–63. 10.1172/jci90593 28665299 PMC5490762

[13] Zeiser R Blazar BR. Acute graft-versus-host disease – biologic process, prevention, and therapy. *N Engl J Med.* (2017) 377:2167–79. 10.1056/NEJMra1609337 29171820 PMC6034180

[14] Tanaka J Imamura M Kasai M Masauzi N Matsuura A Ohizumi H Cytokine gene expression in peripheral blood mononuclear cells during graft-versus-host disease after allogeneic bone marrow transplantation. *Br J Haematol.* (1993) 85:558–65. 10.1111/j.1365-2141.1993.tb03348.x 8136279

[15] Tvedt THA Ersvaer E Tveita AA Bruserud Ø. Interleukin-6 in allogeneic stem cell transplantation: its possible importance for immunoregulation and as a therapeutic target. *Front Immunol.* (2017) 8:667. 10.3389/fimmu.2017.00667 28642760 PMC5462914

[16] O’Shea JJ Ma A Lipsky P. Cytokines and autoimmunity. *Nat Rev Immunol.* (2002) 2:37–45. 10.1038/nri702 11905836

[17] Brenner D Blaser H Mak TW. Regulation of tumour necrosis factor signalling: live or let die. *Nat Rev Immunol.* (2015) 15:362–74. 10.1038/nri3834 26008591

[18] Holler E Kolb HJ Moller A Kempeni J Liesenfeld S Pechumer H Increased serum levels of tumor necrosis factor alpha precede major complications of bone marrow transplantation. *Blood.* (1990) 75:1011–6. 2405918

[19] Schmaltz C Alpdogan O Muriglan SJ Kappel BJ Rotolo JA Ricchetti ET Donor T cell-derived TNF is required for graft-versus-host disease and graft-versus-tumor activity after bone marrow transplantation. *Blood.* (2003) 101:2440–5. 10.1182/blood-2002-07-2109 12424195

[20] Holler E Kolb HJ Mittermuller J Kaul M Ledderose G Duell T Modulation of acute graft-versus-host-disease after allogeneic bone marrow transplantation by tumor necrosis factor alpha (TNF alpha) release in the course of pretransplant conditioning: role of conditioning regimens and prophylactic application of a monoclonal antibody neutralizing human TNF alpha (MAK 195F). *Blood.* (1995) 86:890–9. 7620183

[21] Kitko CL Paczesny S Yanik G Braun T Jones D Whitfield J Plasma elevations of tumor necrosis factor-receptor-1 at day 7 postallogeneic transplant correlate with graft-versus-host disease severity and overall survival in pediatric patients. *Biol Blood Marrow Transplant.* (2008) 14:759–65. 10.1016/j.bbmt.2008.04.002 18541194 PMC2577819

[22] Korngold R Marini JC de Baca ME Murphy GF Giles-Komar J. Role of tumor necrosis factor-alpha in graft-versus-host disease and graft-versus-leukemia responses. *Biol Blood Marrow Transplant.* (2003) 9:292–303. 10.1016/s1083-8791(03)00087-9 12766879

[23] Hamadani M Hofmeister CC Jansak B Phillips G Elder P Blum W Addition of infliximab to standard acute graft-versus-host disease prophylaxis following allogeneic peripheral blood cell transplantation. *Biol Blood Marrow Transplant.* (2008) 14:783–9. 10.1016/j.bbmt.2008.04.006 18541197 PMC4100722

[24] Couriel D Saliba R Hicks K Ippoliti C de Lima M Hosing C Tumor necrosis factor-alpha blockade for the treatment of acute GVHD. *Blood.* (2004) 104:649–54. 10.1182/blood-2003-12-4241 15069017

[25] Couriel DR Saliba R de Lima M Giralt S Andersson B Khouri I A phase III study of infliximab and corticosteroids for the initial treatment of acute graft-versus-host disease. *Biol Blood Marrow Transplant.* (2009) 15:1555–62. 10.1016/j.bbmt.2009.08.003 19896079 PMC4114035

[26] Gatza E Braun T Levine JE Ferrara JL Zhao S Wang T Etanercept plus topical corticosteroids as initial therapy for grade one acute graft-versus-host disease after allogeneic hematopoietic cell transplantation. *Biol Blood Marrow Transplant.* (2014) 20:1426–34. 10.1016/j.bbmt.2014.05.023 24892263 PMC4145722

[27] De Jong CN Saes L Klerk CPW Van der Klift M Cornelissen JJ Broers AEC. Etanercept for steroid-refractory acute graft-versus-host disease: a single center experience. *PLoS One.* (2017) 12:e0187184. 10.1371/journal.pone.0187184 29073260 PMC5658201

[28] van Groningen LF Liefferink AM de Haan AF Schaap NP Donnelly JP Blijlevens NM Combination therapy with inolimomab and etanercept for severe steroid-refractory acute graft-versus-host disease. *Biol Blood Marrow Transplant.* (2016) 22:179–82. 10.1016/j.bbmt.2015.08.039 26386320

[29] Ma CKK García-Cadenas I Fox ML Ai S Nivison-Smith I Milliken ST Poor prognosis in patients with steroid refractory acute graft versus host disease treated with etanercept: a multi-centre analysis. *Bone Marrow Transplant.* (2018) 53:1478–82. 10.1038/s41409-018-0215-4 29795434

[30] Busca A Locatelli F Marmont F Ceretto C Falda M. Recombinant human soluble tumor necrosis factor receptor fusion protein as treatment for steroid refractory graft-versus-host disease following allogeneic hematopoietic stem cell transplantation. *Am J Hematol.* (2007) 82:45–52. 10.1002/ajh.20752 16937391

[31] Wajant H Beilhack A. Targeting regulatory T cells by addressing tumor necrosis factor and its receptors in allogeneic hematopoietic cell transplantation and cancer. *Front Immunol.* (2019) 10:2040. 10.3389/fimmu.2019.02040 31555271 PMC6724557

[32] Jones SA Scheller J Rose-John S. Therapeutic strategies for the clinical blockade of IL-6/gp130 signaling. *J Clin Invest.* (2011) 121:3375–83. 10.1172/jci57158 21881215 PMC3163962

[33] Tanaka T Narazaki M Kishimoto T. IL-6 in inflammation, immunity, and disease. *Cold Spring Harb Perspect Biol.* (2014) 6:a016295. 10.1101/cshperspect.a016295 25190079 PMC4176007

[34] Tawara I Koyama M Liu C Toubai T Thomas D Evers R Interleukin-6 modulates graft-versus-host responses after experimental allogeneic bone marrow transplantation. *Clin Cancer Res.* (2011) 17:77–88. 10.1158/1078-0432.Ccr-10-1198 21047980 PMC3058832

[35] Chen X Das R Komorowski R Beres A Hessner MJ Mihara M Blockade of interleukin-6 signaling augments regulatory T-cell reconstitution and attenuates the severity of graft-versus-host disease. *Blood.* (2009) 114:891–900. 10.1182/blood-2009-01-197178 19491393 PMC2716024

[36] Drobyski WR Pasquini M Kovatovic K Palmer J Douglas Rizzo J Saad A Tocilizumab for the treatment of steroid refractory graft-versus-host disease. *Biol Blood Marrow Transplant.* (2011) 17:1862–8. 10.1016/j.bbmt.2011.07.001 21745454 PMC3716013

[37] Kennedy GA Varelias A Vuckovic S Le Texier L Gartlan KH Zhang P Addition of interleukin-6 inhibition with tocilizumab to standard graft-versus-host disease prophylaxis after allogeneic stem-cell transplantation: a phase 1/2 trial. *Lancet Oncol.* (2014) 15:1451–9. 10.1016/s1470-2045(14)71017-4 25456364

[38] Drobyski WR Szabo A Zhu F Keever-Taylor C Hebert KM Dunn R Tocilizumab, tacrolimus and methotrexate for the prevention of acute graft-versus-host disease: low incidence of lower gastrointestinal tract disease. *Haematologica.* (2018) 103:717–27. 10.3324/haematol.2017.183434 29351985 PMC5865423

[39] Kennedy GA Tey S-K Curley C Butler JP Misra A Subramoniapillai E Results of a phase III double-blind study of the addition of tocilizumab vs. placebo to cyclosporin/methotrexate gvhd prophylaxis after HLA-matched allogeneic stem cell transplantation. *Blood.* (2019) 134(Supp. 1):368–368. 10.1182/blood-2019-126285

[40] McCarthy PL Jr. Williams L Harris-Bacile M Yen J Przepiorka D Ippoliti C A clinical phase I/II study of recombinant human interleukin-1 receptor in glucocorticoid-resistant graft-versus-host disease. *Transplantation.* (1996) 62:626–31. 10.1097/00007890-199609150-00015 8830827

[41] Antin JH Weinstein HJ Guinan EC McCarthy P Bierer BE Gilliland DG Recombinant human interleukin-1 receptor antagonist in the treatment of steroid-resistant graft-versus-host disease. *Blood.* (1994) 84:1342–8. 8049450

[42] Antin JH Weisdorf D Neuberg D Nicklow R Clouthier S Lee SJ Interleukin-1 blockade does not prevent acute graft-versus-host disease: results of a randomized, double-blind, placebo-controlled trial of interleukin-1 receptor antagonist in allogeneic bone marrow transplantation. *Blood.* (2002) 100:3479–82. 10.1182/blood-2002-03-0985 12393661

[43] McCarthy PL Jr. Abhyankar S Neben S Newman G Sieff C Thompson RC Inhibition of interleukin-1 by an interleukin-1 receptor antagonist prevents graft-versus-host disease. *Blood.* (1991) 78:1915–8. 1832996

[44] Jankovic D Ganesan J Bscheider M Stickel N Weber FC Guarda G The Nlrp3 inflammasome regulates acute graft-versus-host disease. *J Exp Med.* (2013) 210:1899–910. 10.1084/jem.20130084 23980097 PMC3782050

[45] Koehn BH Apostolova P Haverkamp JM Miller JS McCullar V Tolar J GVHD-associated, inflammasome-mediated loss of function in adoptively transferred myeloid-derived suppressor cells. *Blood.* (2015) 126:1621–8. 10.1182/blood-2015-03-634691 26265697 PMC4582338

[46] Fujiwara H Docampo MD Riwes M Peltier D Toubai T Henig I Microbial metabolite sensor GPR43 controls severity of experimental GVHD. *Nat Commun.* (2018) 9:3674. 10.1038/s41467-018-06048-w 30201970 PMC6131147

[47] Toubai T Fujiwara H Rossi C Riwes M Tamaki H Zajac C Host NLRP6 exacerbates graft-versus-host disease independent of gut microbial composition. *Nat Microbiol.* (2019) 4:800–12. 10.1038/s41564-019-0373-1 30858572 PMC6689241

[48] Reed MH Shapiro ME Milford EL Carpenter CB Kirkman RL. Interleukin 2 receptor expression on peripheral blood lymphocytes in association with renal allograft rejection. *Transplantation.* (1989) 48:361–6. 10.1097/00007890-198909000-00001 2571202

[49] Kirkman RL Barrett LV Gaulton GN Kelley VE Ythier A Strom TB. Administration of an anti-interleukin 2 receptor monoclonal antibody prolongs cardiac allograft survival in mice. *J Exp Med.* (1985) 162:358–62. 10.1084/jem.162.1.358 3925068 PMC2187689

[50] Abbas AK Trotta E Simeonov D R Marson A Bluestone JA. Revisiting IL-2: biology and therapeutic prospects. *Sci Immunol.* (2018) 3:eaat1482. 10.1126/sciimmunol.aat1482 29980618

[51] Lee SJ Zahrieh D Agura E MacMillan ML Maziarz RT McCarthy PL Jr. Effect of up-front daclizumab when combined with steroids for the treatment of acute graft-versus-host disease: results of a randomized trial. *Blood.* (2004) 104:1559–64. 10.1182/blood-2004-03-0854 15138163

[52] Bordigoni P Dimicoli S Clement L Baumann C Salmon A Witz F Daclizumab, an efficient treatment for steroid-refractory acute graft-versus-host disease. *Br J Haematol.* (2006) 135:382–5. 10.1111/j.1365-2141.2006.06321.x 16984386

[53] Massenkeil G Rackwitz S Genvresse I Rosen O Dorken B Arnold R. Basiliximab is well tolerated and effective in the treatment of steroid-refractory acute graft-versus-host disease after allogeneic stem cell transplantation. *Bone Marrow Transplant.* (2002) 30:899–903. 10.1038/sj.bmt.1703737 12476283

[54] Wang JZ Liu KY Xu LP Liu DH Han W Chen H Basiliximab for the treatment of steroid-refractory acute graft-versus-host disease after unmanipulated HLA-mismatched/haploidentical hematopoietic stem cell transplantation. *Transplant Proc.* (2011) 43:1928–33. 10.1016/j.transproceed.2011.03.044 21693302

[55] Schmidt-Hieber M Fietz T Knauf W Uharek L Hopfenmuller W Thiel E Efficacy of the interleukin-2 receptor antagonist basiliximab in steroid-refractory acute graft-versus-host disease. *Br J Haematol.* (2005) 130:568–74. 10.1111/j.1365-2141.2005.05631.x 16098072

[56] Betts BC Pidala J Kim J Mishra A Nishihori T Perez L IL-2 promotes early Treg reconstitution after allogeneic hematopoietic cell transplantation. *Haematologica.* (2017) 102:948–57. 10.3324/haematol.2016.153072 28104702 PMC5477614

[57] Malek TR Bayer AL. Tolerance, not immunity, crucially depends on IL-2. *Nat Rev Immunol.* (2004) 4:665–74. 10.1038/nri1435 15343366

[58] Taylor PA Lees CJ Blazar BR. The infusion of ex vivo activated and expanded CD4(+)CD25(+) immune regulatory cells inhibits graft-versus-host disease lethality. *Blood.* (2002) 99:3493–9. 10.1182/blood.v99.10.3493 11986199

[59] Hoffmann P Ermann J Edinger M Fathman CG Strober S. Donor-type CD4(+)CD25(+) regulatory T cells suppress lethal acute graft-versus-host disease after allogeneic bone marrow transplantation. *J Exp Med.* (2002) 196:389–99. 10.1084/jem.20020399 12163567 PMC2193938

[60] Edinger M Hoffmann P Ermann J Drago K Fathman CG Strober S CD4+CD25+ regulatory T cells preserve graft-versus-tumor activity while inhibiting graft-versus-host disease after bone marrow transplantation. *Nat Med.* (2003) 9:1144–50. 10.1038/nm915 12925844

[61] Matsuoka K Koreth J Kim HT Bascug G McDonough S Kawano Y Low-dose interleukin-2 therapy restores regulatory T cell homeostasis in patients with chronic graft-versus-host disease. *Sci Transl Med.* (2013) 5:179ra43. 10.1126/scitranslmed.3005265 23552371 PMC3686517

[62] Koreth J Matsuoka K Kim HT McDonough SM Bindra B Alyea EP III Interleukin-2 and regulatory T cells in graft-versus-host disease. *N Engl J Med.* (2011) 365:2055–66. 10.1056/NEJMoa1108188 22129252 PMC3727432

[63] Koreth J Kim HT Jones KT Lange PB Reynolds CG Chammas MJ Efficacy, durability, and response predictors of low-dose interleukin-2 therapy for chronic graft-versus-host disease. *Blood.* (2016) 128:130–7. 10.1182/blood-2016-02-702852 27073224 PMC4937358

[64] Chi H. Regulation and function of mTOR signalling in T cell fate decisions. *Nat Rev Immunol.* (2012) 12:325–38. 10.1038/nri3198 22517423 PMC3417069

[65] Chiang PH Wang L Bonham CA Liang X Fung JJ Lu L Mechanistic insights into impaired dendritic cell function by rapamycin: inhibition of Jak2/Stat4 signaling pathway. *J Immunol.* (2004) 172:1355–63. 10.4049/jimmunol.172.3.1355 14734710

[66] Abouelnasr A Roy J Cohen S Kiss T Lachance S. Defining the role of sirolimus in the management of graft-versus-host disease: from prophylaxis to treatment. *Biol Blood Marrow Transplant.* (2013) 19:12–21. 10.1016/j.bbmt.2012.06.020 22771839

[67] Zeiser R Leveson-Gower DB Zambricki EA Kambham N Beilhack A Loh J Differential impact of mammalian target of rapamycin inhibition on CD4+CD25+Foxp3+ regulatory T cells compared with conventional CD4+ T cells. *Blood.* (2008) 111:453–62. 10.1182/blood-2007-06-094482 17967941 PMC2200823

[68] Antin JH Kim HT Cutler C Ho VT Lee SJ Miklos DB Sirolimus, tacrolimus, and low-dose methotrexate for graft-versus-host disease prophylaxis in mismatched related donor or unrelated donor transplantation. *Blood.* (2003) 102:1601–5. 10.1182/blood-2003-02-0489 12730113

[69] Cutler C Antin JH. Sirolimus for GVHD prophylaxis in allogeneic stem cell transplantation. *Bone Marrow Transplant.* (2004) 34:471–6. 10.1038/sj.bmt.1704604 15273708

[70] Torlen J Ringden O Garming-Legert K Ljungman P Winiarski J Remes K A prospective randomized trial comparing cyclosporine/methotrexate and tacrolimus/sirolimus as graft-versus-host disease prophylaxis after allogeneic hematopoietic stem cell transplantation. *Haematologica.* (2016) 101:1417–25. 10.3324/haematol.2016.149294 27662016 PMC5394879

[71] Sandmaier BM Kornblit B Storer BE Olesen G Maris MB Langston AA Addition of sirolimus to standard cyclosporine plus mycophenolate mofetil-based graft-versus-host disease prophylaxis for patients after unrelated non-myeloablative haemopoietic stem cell transplantation: a multicentre, randomised, phase 3 trial. *Lancet Haematol.* (2019) 6:e409–18. 10.1016/s2352-3026(19)30088-2 31248843 PMC6686903

[72] Xhaard A Launay M Sicre de Fontbrune F Michonneau D Sutra Del Galy A Coman T A monocentric study of steroid-refractory acute graft-versus-host disease treatment with tacrolimus and mTOR inhibitor. *Bone Marrow Transplant.* (2020) 55:86–92. 10.1038/s41409-019-0633-y 31413313

[73] Schroeder MA Choi J Staser K DiPersio JF. The role of janus kinase signaling in graft-versus-host disease and graft versus leukemia. *Biol Blood Marrow Transplant.* (2018) 24:1125–34. 10.1016/j.bbmt.2017.12.797 29289756 PMC5993569

[74] Choi J Cooper ML Alahmari B Ritchey J Collins L Holt M Pharmacologic blockade of JAK1/JAK2 reduces GvHD and preserves the graft-versus-leukemia effect. *PLoS One.* (2014) 9:e109799. 10.1371/journal.pone.0109799 25289677 PMC4188578

[75] Spoerl S Mathew NR Bscheider M Schmitt-Graeff A Chen S Mueller T Activity of therapeutic JAK 1/2 blockade in graft-versus-host disease. *Blood.* (2014) 123:3832–42. 10.1182/blood-2013-12-543736 24711661

[76] Carniti C Gimondi S Vendramin A Recordati C Confalonieri D Bermema A Pharmacologic inhibition of JAK1/JAK2 signaling reduces experimental murine acute GVHD while preserving GVT effects. *Clin Cancer Res.* (2015) 21:3740–9. 10.1158/1078-0432.Ccr-14-2758 25977345

[77] Jagasia M Zeiser R Arbushites M Delaite P Gadbaw B Bubnoff NV. Ruxolitinib for the treatment of patients with steroid-refractory GVHD: an introduction to the REACH trials. *Immunotherapy.* (2018) 10:391–402. 10.2217/imt-2017-0156 29316837

[78] Chen Y-B Arbushites M Delaite P Yan Y Zeiser R. Trial in progress: Gravitas-301, a randomized, double-blind phase 3 study of itacitinib or placebo with corticosteroids (CS) for the first-line treatment of patients with acute Gvhd (aGVHD). *Biol Blood Marrow Transplant.* (2018) 24:S208–9. 10.1016/j.bbmt.2017.12.177

[79] Carrell RW Lomas DA. Alpha1-antitrypsin deficiency–a model for conformational diseases. *N Engl J Med.* (2002) 346:45–53. 10.1056/NEJMra010772 11778003

[80] Jonigk D Al-Omari M Maegel L Muller M Izykowski N Hong J Anti-inflammatory and immunomodulatory properties of alpha1-antitrypsin without inhibition of elastase. *Proc Natl Acad Sci USA.* (2013) 110:15007–12. 10.1073/pnas.1309648110 23975926 PMC3773761

[81] Bergin DA Reeves EP Hurley K Wolfe R Jameel R Fitzgerald S The circulating proteinase inhibitor alpha-1 antitrypsin regulates neutrophil degranulation and autoimmunity. *Sci Transl Med.* (2014) 6:217ra1. 10.1126/scitranslmed.3007116 24382893

[82] Marcondes AM Karoopongse E Lesnikova M Margineantu D Welte T Dinarello CA α-1-Antitrypsin (AAT)–modified donor cells suppress GVHD but enhance the GVL effect: a role for mitochondrial bioenergetics. *Blood.* (2014) 124:2881–91. 10.1182/blood-2014-04-570440 25224412 PMC4215316

[83] Marcondes AM Li X Tabellini L Bartenstein M Kabacka J Sale GE Inhibition of IL-32 activation by alpha-1 antitrypsin suppresses alloreactivity and increases survival in an allogeneic murine marrow transplantation model. *Blood.* (2011) 118:5031–9. 10.1182/blood-2011-07-365247 21900190 PMC3208308

[84] Tawara I Sun Y Lewis EC Toubai T Evers R Nieves E Alpha-1-antitrypsin monotherapy reduces graft-versus-host disease after experimental allogeneic bone marrow transplantation. *Proc Natl Acad Sci USA.* (2012) 109:564–9. 10.1073/pnas.1117665109 22203983 PMC3258603

[85] Marcondes AM Hockenbery D Lesnikova M Dinarello CA Woolfrey A Gernsheimer T Response of steroid-refractory Acute GVHD to alpha1-antitrypsin. *Biol Blood Marrow Transplant.* (2016) 22:1596–601. 10.1016/j.bbmt.2016.05.011 27223109

[86] Magenau JM Goldstein SC Peltier D Soiffer RJ Braun T Pawarode A alpha1-Antitrypsin infusion for treatment of steroid-resistant acute graft-versus-host disease. *Blood.* (2018) 131:1372–9. 10.1182/blood-2017-11-815746 29437593 PMC5865235

[87] Narita T Weinert BT Choudhary C. Functions and mechanisms of non-histone protein acetylation. *Nat Rev Mol Cell Biol.* (2019) 20:156–74. 10.1038/s41580-018-0081-3 30467427

[88] Shakespear MR Halili MA Irvine KM Fairlie DP Sweet MJ. Histone deacetylases as regulators of inflammation and immunity. *Trends Immunol.* (2011) 32:335–43. 10.1016/j.it.2011.04.001 21570914

[89] Brogdon JL Xu Y Szabo SJ An S Buxton F Cohen D Histone deacetylase activities are required for innate immune cell control of Th1 but not Th2 effector cell function. *Blood.* (2007) 109:1123–30. 10.1182/blood-2006-04-019711 17008546

[90] Bosisio D Vulcano M Del Prete A Sironi M Salvi V Salogni L Blocking TH17-polarizing cytokines by histone deacetylase inhibitors in vitro and in vivo. *J Leukoc Biol.* (2008) 84:1540–8. 10.1189/jlb.0708401 18780875 PMC2614600

[91] Reddy P Sun Y Toubai T Duran-Struuck R Clouthier SG Weisiger E Histone deacetylase inhibition modulates indoleamine 2,3-dioxygenase-dependent DC functions and regulates experimental graft-versus-host disease in mice. *J Clin Invest.* (2008) 118:2562–73. 10.1172/jci34712 18568076 PMC2430497

[92] Reddy P Maeda Y Hotary K Liu C Reznikov LL Dinarello CA Histone deacetylase inhibitor suberoylanilide hydroxamic acid reduces acute graft-versus-host disease and preserves graft-versus-leukemia effect. *Proc Natl Acad Sci USA.* (2004) 101:3921–6. 10.1073/pnas.0400380101 15001702 PMC374345

[93] Choi S Reddy P. HDAC inhibition and graft versus host disease. *Mol Med.* (2011) 17:404–16. 10.2119/molmed.2011.00007 21298214 PMC3105142

[94] Choi SW Braun T Chang L Ferrara JL Pawarode A Magenau JM Vorinostat plus tacrolimus and mycophenolate to prevent graft-versus-host disease after related-donor reduced-intensity conditioning allogeneic haemopoietic stem-cell transplantation: a phase 1/2 trial. *Lancet Oncol.* (2014) 15:87–95. 10.1016/s1470-2045(13)70512-6 24295572 PMC4103793

[95] Choi SW Braun T Henig I Gatza E Magenau J Parkin B Vorinostat plus tacrolimus/methotrexate to prevent GVHD after myeloablative conditioning, unrelated donor HCT. *Blood.* (2017) 130:1760–7. 10.1182/blood-2017-06-790469 28784598 PMC5639486

[96] Perez L Fernandez H Horna P Riches M Locke F Field T Phase I trial of histone deacetylase inhibitor panobinostat in addition to glucocorticoids for primary therapy of acute graft-versus-host disease. *Bone Marrow Transplant.* (2018) 53:1434–44. 10.1038/s41409-018-0163-z 29670210 PMC7771280

[97] Ciechanover A. The ubiquitin-proteasome proteolytic pathway. *Cell.* (1994) 79:13–21. 10.1016/0092-8674(94)90396-4 7923371

[98] Chen ZJ. Ubiquitin signalling in the NF-kappaB pathway. *Nat Cell Biol.* (2005) 7:758–65. 10.1038/ncb0805-758 16056267 PMC1551980

[99] Manasanch EE Orlowski RZ. Proteasome inhibitors in cancer therapy. *Nat Rev Clin Oncol.* (2017) 14:417–33. 10.1038/nrclinonc.2016.206 28117417 PMC5828026

[100] Sunwoo JB Chen Z Dong G Yeh N Crowl Bancroft C Sausville E Novel proteasome inhibitor PS-341 inhibits activation of nuclear factor-kappa B, cell survival, tumor growth, and angiogenesis in squamous cell carcinoma. *Clin Cancer Res.* (2001) 7:1419–28. 11350913

[101] Palombella VJ Conner EM Fuseler JW Destree A Davis JM Laroux FS Role of the proteasome and NF-kappaB in streptococcal cell wall-induced polyarthritis. *Proc Natl Acad Sci USA.* (1998) 95:15671–6. 10.1073/pnas.95.26.15671 9861028 PMC28102

[102] Vodanovic-Jankovic S Hari P Jacobs P Komorowski R Drobyski WR. NF-kappaB as a target for the prevention of graft-versus-host disease: comparative efficacy of bortezomib and PS-1145. *Blood.* (2006) 107:827–34. 10.1182/blood-2005-05-1820 16174760 PMC1895627

[103] Blanco B Perez-Simon JA Sanchez-Abarca LI Carvajal-Vergara X Mateos J Vidriales B Bortezomib induces selective depletion of alloreactive T lymphocytes and decreases the production of Th1 cytokines. *Blood.* (2006) 107:3575–83. 10.1182/blood-2005-05-2118 16282346

[104] Al-Homsi AS Feng Y Duffner U Al Malki MM Goodyke A Cole K Bortezomib for the prevention and treatment of graft-versus-host disease after allogeneic hematopoietic stem cell transplantation. *Exp Hematol.* (2016) 44:771–7. 10.1016/j.exphem.2016.05.005 27224851

[105] Sun K Welniak LA Panoskaltsis-Mortari A O’Shaughnessy MJ Liu H Barao I Inhibition of acute graft-versus-host disease with retention of graft-versus-tumor effects by the proteasome inhibitor bortezomib. *Proc Natl Acad Sci USA.* (2004) 101:8120–5. 10.1073/pnas.0401563101 15148407 PMC419567

[106] Pai CC Hsiao HH Sun K Chen M Hagino T Tellez J Therapeutic benefit of bortezomib on acute graft-versus-host disease is tissue specific and is associated with interleukin-6 levels. *Biol Blood Marrow Transplant.* (2014) 20:1899–904. 10.1016/j.bbmt.2014.07.022 25064746 PMC4254314

[107] Sun K Wilkins DE Anver MR Sayers TJ Panoskaltsis-Mortari A Blazar BR Differential effects of proteasome inhibition by bortezomib on murine acute graft-versus-host disease (GVHD): delayed administration of bortezomib results in increased GVHD-dependent gastrointestinal toxicity. *Blood.* (2005) 106:3293–9. 10.1182/blood-2004-11-4526 15961519 PMC1895334

[108] Liang Y Ma S Zhang Y Wang Y Cheng Q Wu Y IL-1beta and TLR4 signaling are involved in the aggravated murine acute graft-versus-host disease caused by delayed bortezomib administration. *J Immunol.* (2014) 192:1277–85. 10.4049/jimmunol.1203428 24363427

[109] Koreth J Stevenson KE Kim HT McDonough SM Bindra B Armand P Bortezomib-based graft-versus-host disease prophylaxis in HLA-mismatched unrelated donor transplantation. *J Clin Oncol.* (2012) 30:3202–8. 10.1200/jco.2012.42.0984 22869883 PMC3434979

[110] Koreth J Kim HT Lange PB Poryanda SJ Reynolds CG Rai SC Bortezomib-based immunosuppression after reduced-intensity conditioning hematopoietic stem cell transplantation: randomized phase II results. *Haematologica.* (2018) 103:522–30. 10.3324/haematol.2017.176859 29326124 PMC5830392

[111] Al-Homsi AS Goodyke A Cole K Muilenburg M McLane M Abdel-Mageed S Ixazomib suppresses human dendritic cell and modulates murine graft-versus-host disease in a schedule-dependent fashion. *Exp Hematol.* (2017) 48:50–7. 10.1016/j.exphem.2016.12.002 28007655

[112] Ritter AT Angus KL Griffiths GM. The role of the cytoskeleton at the immunological synapse. *Immunol Rev.* (2013) 256:107–17. 10.1111/imr.12117 24117816 PMC4312978

[113] Stinchcombe JC Majorovits E Bossi G Fuller S Griffiths GM. Centrosome polarization delivers secretory granules to the immunological synapse. *Nature.* (2006) 443:462–5. 10.1038/nature05071 17006514

[114] Huse M Lillemeier BF Kuhns MS Chen DS Davis MM. T cells use two directionally distinct pathways for cytokine secretion. *Nat Immunol.* (2006) 7:247–55. 10.1038/ni1304 16444260

[115] Kanapin A Batalov S Davis MJ Gough J Grimmond S Kawaji H Mouse proteome analysis. *Genome Res.* (2003) 13:1335–44. 10.1101/gr.978703 12819131 PMC403658

[116] Huh WK Falvo JV Gerke LC Carroll AS Howson RW Weissman JS Global analysis of protein localization in budding yeast. *Nature.* (2003) 425:686–91. 10.1038/nature02026 14562095

[117] Novick P Field C Schekman R. Identification of 23 complementation groups required for post-translational events in the yeast secretory pathway. *Cell.* (1980) 21:205–15. 10.1016/0092-8674(80)90128-2 6996832

[118] Barlowe C Orci L Yeung T Hosobuchi M Hamamoto S Salama N COPII: a membrane coat formed by Sec proteins that drive vesicle budding from the endoplasmic reticulum. *Cell.* (1994) 77:895–907. 10.1016/0092-8674(94)90138-4 8004676

[119] Schlacht A Dacks JB. Unexpected ancient paralogs and an evolutionary model for the COPII coat complex. *Genome Biol Evol.* (2015) 7:1098–109. 10.1093/gbe/evv045 25747251 PMC4419792

[120] Kim S Khoriaty R Valanparambil R Wu J Taylor A Fujiwara H Targeting Sec23b in COPII vesicles regulates T cell immunity. *Blood.* (2018) 132(Suppl. 1):859–859. 10.1182/blood-2018-99-111628

